# Successful Nicotine Intake in Medical Assisted Use of E-Cigarettes: A Pilot Study

**DOI:** 10.3390/ijerph120707638

**Published:** 2015-07-08

**Authors:** Roberta Pacifici, Simona Pichini, Silvia Graziano, Manuela Pellegrini, Giuseppina Massaro, Fabio Beatrice

**Affiliations:** 1 Drug Abuse and Doping Unit, Department of Therapeutic Research and Medicines Evaluation National Institute of Health, Roma 00161, Italy; E-Mails: roberta.pacifici@iss.it (R.P.); silviagrzn@gmail.com (S.G.); manuela.pellegrini@iss.it (M.P.); 2 Ospedale San Giovanni Bosco, ASL TO2 Torino 10154, Italy; E-Mails: fabio.beatrice@tin.it (F.B.); massaro.giuseppina@yahoo.it (G.M.)

**Keywords:** smoking cigarette, cotinine, *trans*-3’-hydroxycotinine, electronic cigarette, tobacco harm reduction

## Abstract

The electronic cigarette (e-cig) has gained popularity as an aid in smoking cessation programs mainly because it maintains the gestures and rituals of tobacco smoking. However, it has been shown in inexperienced e-cig users that ineffective nicotine delivery can cause tobacco craving that could be responsible for unsuccessful smoking reduction/cessation. Moreover, the incorrect use of an e-cig could also led to potential nicotine overdosage and intoxication. Medically assisted training on the proper use of an e-cig plus behavioral support for tobacco dependence could be a pivotal step in avoiding both issues. We performed an eight-month pilot study of adult smokers who started e-cig use after receiving a multi-component medically assisted training program with monitoring of nicotine intake as a biomarker of correct e-cig use. Participants were tested during follow-up for breath carbon monoxide (CO), plasma cotinine and *trans*-3’-hydroxycotinine, and number of tobacco cigarettes smoked. At the end of the first, fourth, and eighth month of follow-up, 91.1, 73.5, and 76.5% of participants respectively were e-cig users (‘only e-cig’ and ‘dual users’). They showed no significant variation in plasma cotinine and *trans*-3’-hydroxycotinine with respect to the start of the study when they smoked only tobacco cigarettes, but a significant reduction in breath CO. The proposed medically assisted training program of e-cig use led to a successful nicotine intake, lack of typical cigarette craving and overdosage symptoms and a significant decrease in the biomarker of cigarette combustion products.

## 1. Introduction

During the past few years electronic cigarettes (e-cigs) have gained popularity, primarily among smokers who want to reduce the risks of tobacco smoking. The popularity of e-cigs is linked to their perceived safety since they completely avoid the combustion of organic material (tobacco), and hence of the toxic and carcinogenic chemicals that are present in cigarette smoke; however long-term safety is currently unknown, and concerns have been raised about the potential risks of nicotine poisoning [1].

Several studies have evaluated relationships between e-cig use and smoking reduction and cessation, but the results are still controversial [2,3,4]. The main drawback observed in several clinical studies seems to be the ineffective nicotine delivery in inexperienced e-cig users as shown by low or even negligible measurements of nicotine (NIC) and/or its major metabolite cotinine (COT) in cig users compared to those obtained after smoking tobacco cigarettes [5]. Therefore, the incorrect use of e-cigs could be responsible for tobacco craving, leading to unsuccessful smoking reduction/cessation. Moreover, overpuffing on an e-cig could potentially lead to nicotine overdosage and intoxication [6,7,8]. Medically assisted training on the proper use of e-cigs could be a pivotal step in avoiding both issues, that could result in higher success rates in smoking reduction/cessation. We performed an eight-month pilot study in adult smokers who received a medically assisted training program plus behavioral support for tobacco dependence. The study evaluated e-cig use, smoking reduction and/or smoking abstinence, levels of carbon monoxide in exhaled breath as a biomarker of cigarette combustion products and plasma COT and *trans-*3’-hydroxycotinine (3-HCOT), as biomarkers of nicotine intake. 

## 2. Experimental Section 

### 2.1. Study Design and Medical Assisted Training on E-Cig

Adult smokers unwilling to quit smoking tobacco cigarettes and who have never tried a quit smoking protocol and/or have refused any smoking cessation treatment were recruited by word of mouth for the uncontrolled pre-post pilot study, carried out at the anti-smoking detoxification Centre at San Giovanni Bosco Hospital (Torino, Italy) that supplies individualized smoking cessation treatments since 2000 to an average of 300 smokers per year. The study was approved by the Istituto Superiore di Sanità ethical committee (CE 14/418) and carried out in compliance with the Helsinki Declaration. After a first meeting to inform about the study design, written informed consent for the involvement in the study was obtained from all participants. At the baseline, participants were investigated about their age, weight, sport habit, physical status, social class, education and smoking history; this has included years as a smoker, daily number of cigarettes, and cigarette brand. Additionally, participants were scored for their level of nicotine dependence by the Fagestrom Test of Nicotine Dependence (FTND). Levels of carbon monoxide (CO) in exhaled breath as biomarker of absorption of cigarette combustion products were measured using the MicroMedical micro Co monitor (SensorMedics Italia srl, Milan, Italy) and blood samples were collected to determine principal nicotine metabolites: COT and 3-HCOT. Participants were given a couple of commercially available e-cigs (AVATAR device, Battery 550 mAh/3.9 V, W: 7.8, cartomizer with 2, 2 ohm resistance, tank capacity 1.5 mL, temperature of the aerosol: 55/65 degrees), two different chargers for each e-cig and PUFFIT e-liquids with nicotine content matching the individual nicotine daily intake and tobacco and/or other flavours freely chosen by each participant. Information about general working principles, safety and risks of e-cigs were given to the participants by Dr. Beatrice, the chief of the Detoxification Centre and his medical and nursing staff, together with medically assisted face-to-face training on how to correctly use the device to absorb nicotine vapor. This medical assisted training (Jacobacci & Partners, trademark number 013388186) has been developed by the same chief of the Detoxification Centre at San Giovanni Bosco Hospital and colleagues as a translational program from tobacco cigarette to e-cig for smokers unwilling to quit smoking especially because of the gestures and rituals of cigarette smoking, known to be linked with specific feelings (*i.e.*, relax or stress circumstances). Classical smoking cessation treatments (*i.e.*, Nicotine Replacement Therapy, varenicline or bupropion) cannot replace the rituals associated with the act of smoking, and when the smoker stops smoking the need for the ritual sometimes still exists and can cause relapses; so classical treatments are generally strongly refused by smokers who do not want to stop smoking tobacco cigarettes [9,10].

Specifically, in the first week participants were trained to correctly use the e-cig using an e-liquid without nicotine, and were helped to focus on the circumstances in which they smoked. During the second and third weeks, participants started to use their own e-cig charged with e-liquid containing the personal nicotine dosage, and were encouraged to use it as a substitute for tobacco cigarettes that they previously identified as being less necessary. The fourth week was dedicated to reinforce the substitution of all the tobacco cigarettes with the e-cig throughout the day. Furthermore, participants were assisted during all the study period (including training weeks) at any time of the day by Dr. Beatrice, and his medical and nursing staff, with any possible problem a related to e-cigs, both through established follow-up group sessions, and also through an instant messaging application for smartphones (Whatsapp Inc.) which included all the participants and the trainers in a chat group. Although participants were encouraged to use the e-cig, they were also permitted to smoke their favorite brand of tobacco cigarettes during this study. At the first, fourth, and eighth month follow-up visits, participants were tested again for CO levels in exhaled breath, and blood samples were collected to determine COT and 3-HCOT concentration. Finally, participants were asked to report the daily number of cigarettes smoked at the time of the visit and any adverse events experienced. The data were analysed using 2-tailed statistical tests, and *p* values < 0.05 were considered significant.

### 2.2. Blood Analysis

Blood samples underwent a liquid chromatography-tandem mass spectrometry (LC/MS/MS) analysis of principal nicotine metabolites: cotinine (COT) and *trans*-3’-hydroxycotinine (3-HCOT): 10 μL of internal standard (1’-*N*-ethylnorcotinine, NENC, Sigma Aldrich, Milano, Italy) and 2 mL of a chloroform/isopropyl alcohol solution were added to 500 μL of each plasma sample. After centrifugation at 3000 rpm for 3 min, the organic phase was collected and evaporated under a nitrogen stream at ambient temperature. Samples were redissolved in 100 μL LC mobile phase (water/methanol/acetonitrile, 70:25:5, v/v/v). A 20 μL volume was injected into the LC/MS/MS system applying a methodology reported elsewhere [11].

## 3. Results and Discussion

Thirty-four participants (52.9% men, 47.1% women) unwilling to quit smoking were recruited for the study. Participants were aged 18–63 years old (40.6 ± 13.5, M ± SD), smoked ≥ 10 cigarettes per day (CPD, (21.5 ± 9.0, M ± SD) for at least 4 years (range 4–41 years, 22.0 ± 11.0 M ± SD) and the FTND indicated a moderate-strong nicotine dependence. The majority of the participants had an average education level (median and high school, 88.2%) and a profession in the semi-skilled category (61.8%) (Table 1).

**Table 1:** Summary of participants characteristics.

	Participants (n)	Men (%)	Women (%)
	34	52.9	47.1
Age (years, M ± SD *)	40.6 ± 12.1	40.7 ± 12.6	40.6 ± 11.9
**Weight (kg, M ± SD)**	70.9 ± 13.5	76.4 ± 10.4	64.8 ± 14.1
**Cigarettes per Day (M ± SD)**	21.5 ± 9.0	21.1 ± 10.8	21.9 ± 13.9
**Years as Smoker (M ± SD)**	22.0 ± 11.0	23.4 ± 11.2	20.5 ± 11.2
**Education (%)**			
**Primary School**	2.9	2.9	0.0
**Median School**	44.1	20.6	23.6
**High School**	44.1	32.4	11.8
**University Degree**	8.8	2.9	5.9
**Profession (%)**			
**Unskilled**	11.8	11.1	12.5
**Semi Skilled**	61.8	55.6	68.8
**Skilled**	17.6	16.7	18.8
**Manager**	8.8	16.7	0.0

* M ± SD: Mean and Standard Deviation.

At the end of the first month of follow-up (T1), participants could be classified in three categories; only e-cig users, dual users, and only cigarette users (73.5%, 17.6%, and 8.8% respectively).

**Table 2:** First month follow-up of medical assisted use of e-cigarette.

	Only E-Cig	Dual Use	Only Cigarette
**Participants (n = 34)**	73.5% (n = 25)	17.6% (n = 6)	8.8% (n = 3)
Age (years, M ± SD *)	40.4 ± 11.5	41.5 ± 17.3	40.7 ± 8.3
**Fagestrom Test ( M ± SD)**	5.3 ± 2.2	5.0 ± 2.0	5.3 ± 2.5
**Cigarettes at T0 (M ± SD)**	19.8 ± 5.7	23.3 ± 6.1	31.7 ± 25.7
**Cigarettes at T1 (M ± SD)**	0	2.3 ± 1.5	19.0 ± 18.5
***p*-test**	<0.0001	0.0002	0.5
**Breath CO at T0 (M ± SD)**	2.4 ± 1.3	2.5 ± 0.6	4.6 ± 1.3
**Breath CO at T1 (M ± SD)**	0.3 ± 0.1	0.5 ± 0.3	4.5 ± 2.5
***p*-test**	<0.0001	<0.0001	0.9
**Plasma COT at T0 (M ± SD)**	152.3 ± 92.3	115.1 ± 65.8	279.8 ± 15.6
**Plasma COT at T1 (M ± SD)**	156.1 ± 108.6	151.5 ± 71.0	210.6 ± 88.7
***p*-test**	0.9	0.4	0.3
**Plasma 3-HCOT at T0 (M ± SD)**	49.0 ± 24.8	41.6 ± 37.7	93.0 ± 63.2
**Plasma 3-HCOT at T1 (M ± SD)**	45.5 ± 31.5	56.5 ± 34.3	99.5 ± 52.0
***p*-test**	0.7	0.5	0.9

* M ± SD: Mean ± Standard Deviation.

A summary of their characteristics such as the values of breath CO, plasma COT and 3-HCOT during the study is given in Table 2. E-cig users showed a statistically significant reduction of breath CO whereas no differences were detected in COT, and 3-HCOT plasma concentrations with respect to the start of the study. Dual users significantly reduced the number of cigarettes and the value of breath CO. Also in this last group no significant differences were detected in plasma COT and 3-HCOT. Only cigarette users did not show any significant variation in the number of smoked cigarettes, value of breath CO and plasma COT and 3-HCOT concentrations**.**

At the end of the fourth month of follow up (T4) 40% (n = 10) of only e-cig users resumed cigarette smoking, increasing the percentage of dual and only cigarette users to 23.5% and 26.5%, respectively (Table 3). However, dual and only cigarette users still showed a significant reduction in the number of daily cigarettes since the start of the study and, at the same time, in the value of breath CO. Again, no significant differences were detected in COT and 3-HCOT plasma concentration in all three users groups.

**Table 3:** Fourth month follow-up of medical assisted use of e-cigarette.

	Only E-Cig	Dual Use	Only Cigarette
**Participants (n = 34)**	50 (n = 17)	23.5 (n = 8)	26.5 (n = 9)
**Age (years, M ± SD)**	40.8 ± 12.6	39.6 ± 11.3	41.3 ± 13.1
**Fagestrom Test (M ± SD)**	5.1 ± 2.1	5.6 ± 2.7	5.3 ± 1.9
**Cigarettes at T0 (M ± SD)**	18.8 ± 5.2	21.9 ± 6.5	26.1 ± 14.3
**Cigarettes at T4 (M ± SD)**	0	2.4 ± 1.4	5.7 ± 3.4
***p*-test**	<0.0001	<0.0001	0.001
**Breath CO at T0 (M ± SD)**	2.1 ± 1.2	2.9 ± 1.5	3.3 ± 1.3
**Breath CO at T4 (M ± SD)**	0.3 ± 0.2	0.7 ± 0.1	1.7 ± 0.8
***p*-test**	<0.0001	0.001	0.005
**Plasma COT at T0 (M ± SD)**	139.3 ± 96.7	148.8 ± 88.6	197.5 ± 85.1
**Plasma COT at T4 (M ± SD)**	98.8 ± 79.2	127.3 ± 77.3	143.5 ± 63.6
***p*-test**	0.2	0.6	0.1
**Plasma 3-HCOT at T0 (M ± SD)**	42.9 ± 27.5	51.8 ± 27.4	67.7 ± 43.1
**Plasma 3-HCOt at T4 (M ± SD)**	29.3 ± 24.4	29.4 ± 27.5	35.5 ± 21.6
***p*-test**	0.1	0.1	0.06

* M ± SD: Mean ± Standard Deviation.

At the end of the eighth month follow up (T8) there were no major changes in the only e-cig, dual, and only cigarettes groups (Table 4). As described above, no significant differences were detected in COT and 3-HCOT plasma concentration, and a significant reduction in breath CO and in the number of smoked cigarette was still detectable in all three groups. During the training period and eight month follow-up, none of the participants became both nicotine and tobacco free, namely a complete non-nicotine user.

The use of e-cigs as an aid to smoking reduction/cessation is still controversial [2,3,4]. One major issue appears to be the ineffective absorption of nicotine, as shown in some studies by the negligible concentrations of plasma COT if compared to those obtained from tobacco smoking, most typically associated with the use of first generation e-cigs and/-or the incorrect use of the device [5]. In the recent ECLAT study on smokers unwilling to quit, the employment of a first generation device with training on correct use led to a complete abstinence from tobacco smoking in 10.7% and 8.7% at week-12 and week-52, respectively [12]. Concurrently, saliva COT levels were well below the concentration threshold representative of regular smokers or experienced e-cig users. Moreover approximately 40% of the participants failed to attend their final follow-up visit, and the failure to complete the study was likely to be due to a number of technical issues (e.g., e-cig malfunctions) declared by the participants [12]. 

**Table 4:** Eighth month follow-up of medical assisted use of e-cigarette.

	Only E-Cig	Dual Use	Only Cigarette
**Participants (n = 34)**	52.9 (n = 18)	23.5 (n = 8)	23.5 (n = 8)
**Age (years, M ± SD)**	42.8 ± 11.1	35.5 ± 12.5	40.9 ± 13.8
**Fagestrom Test (M ± SD)**	4.9 ± 2.1	5.8 ± 2.6	5.5 ± 2.0
**Cigarettes at T0 (M ± SD)**	18.6 ± 5.1	22.5 ± 6.0	26.9 ± 15.1
**Cigarettes at T8 (M ± SD)**	0	5.3 ± 2.5	7.7 ± 6.3
***p*-test**	<0.0001	<0.0001	<0.05
**Breath CO at T0 (M ± SD)**	2.1 ± 1.2	2.9 ± 1.5	3.5 ± 1.3
**Breath CO at T8 (M ± SD)**	0.3 ± 0.1	0.8 ± 0.3	1.9 ± 1.5
***p*-test**	<0.0001	0.001	<0.05
**Plasma COT at T0 (M ± SD)**	153.3 ± 96.1	139.9 ± 95.5	182.3 ± 88.3
**Plasma COT at T8 (M ± SD)**	114.4 ± 75.5	164.9 ± 91.6	169.6 ± 76.1
***p*-test**	0.2	0.6	0.7
**Plasma 3-HCOT at T0 (M ± SD)**	47.0 ± 26.6	42.4 ± 26.4	71.1 ± 46.0
**Plasma 3-HCOt at T8 (M ± SD)**	36.5 ± 29.0	33.9 ± 15.6	57.7 ± 36.8
***p*-test**	0.3	0.5	0.5

* M ± SD: Mean ± Standard Deviation.

Furthermore, a recent systematic review on sixteen clinical studies on e-cigs highlights that even if second generation e-cigs are capable of delivering a similar amount of nicotine as traditional tobacco cigarettes, inexperienced users are not able to achieve systemic NIC and/or COT concentrations similar to those produced from traditional cigarettes [5]. 

Conversely, Adriaens *et al*. showed that giving clear instructions on the use of a second generation e-cig in a group of smokers, unwilling to quit and who had never tried e-cigs, led to an immediate and strong craving reduction with only little withdrawal symptoms after e-cig abstinence for four hours. Accordingly, saliva COT levels did not show any difference between only e-cig users, dual smokers and only tobacco smokers and exhaled breath CO levels decreased in all the three groups of smokers following the reduction/abstinence from tobacco cigarettes [13].

These data are in agreement with the results of the present study. Both e-cig users and dual users showed no significant variations in COT and 3-HCOT plasma concentrations at first, fourth and eighth month follow ups, meaning that they were obtaining a similar nicotine intake as when smoking only tobacco cigarettes, and a significant reduction in the absorption of cigarette combustion products was demonstrated by a significant decrease in breath CO. At the end of the T4 follow-up, the reintroduction of tobacco cigarettes was reported by 40% of only e-cig users at T1; however the number of smoked tobacco cigarettes was so low that it did not increase breath CO values. Data at the end of T8 follow up showed only a slight variation in the number of participants in the only e-cig and only cig groups. A significant reduction in the breath CO compared to T0 was still present, even when the 37.5% of only cigarette users reported an increased number of daily cigarettes compared to the fourth month of follow up. Plasma concentration of COT and 3-HCOT did not show significant variation in all three groups from the start of the study, consolidating our previous data. It should be noted that at the end of T8 follow-up about 53% of participants were still abstinent from tobacco cigarettes (only e-cig users); this cessation rate is higher than the 21% obtained by Adriaens and colleagues at the same time interval [13]. In light of this result, it could be considered that the technical, behavioural, and psychological support offered in our medically assisted training was able to offer a quicker and efficient intervention to help the participants.

A criticism that could be raised for the present study is the absence of a control group of smokers not undergoing medically assisted training program before starting e-cigs in order to demonstrate the efficacy of the training. However, the group of smokers who tried and immediately refused to start the e-cig can be considered a “control” group.

## 4. Conclusions 

Although this study presents the important limitation of small number of participants, the promising results suggest that the proposed transitional program from tobacco cigarette by a patented multicomponent medically assisted training program on e-cig use assists the reduction of cigarette consumption in smokers not intending to quit, by assuring successful nicotine intake, avoiding nicotine overdosage and intoxication and absorption of cigarette combustion products, while considering the behavioural components of tobacco dependence. However, a more extended fully-powered randomized controlled trial with more participants and a control group not following the transitional program is necessary to strengthen these data. 
